# Comparison of health‐related quality of life and cosmetic outcome between traditional gasless trans‐axillary endoscopic thyroidectomy and modified gasless trans‐axillary endoscopic thyroidectomy for patients with papillary thyroid microcarcinoma

**DOI:** 10.1002/cam4.6258

**Published:** 2023-06-19

**Authors:** Deenraj Kush Dhoomun, HuiLan Cai, Ning Li, YanHuan Qiu, XingRui Li, XiaoPeng Hu, WenZhuang Shen

**Affiliations:** ^1^ Department of Thyroid and Breast Surgery, Tongji Hospital, Tongji Medical College Huazhong University of Science and Technology (HUST) Wuhan China

**Keywords:** laparoscopic surgery, quality of life, therapeutic results, thyroid cancer

## Abstract

**Background:**

Gasless trans‐axillary endoscopic thyroidectomy (GTET) has been proved to provide better cosmetic results; however, it has limitations as dissection of central neck lymph nodes is difficult. We developed a modified approach (MGTET‐modified GTET) and compared it with the traditional one in terms of patients' health‐related quality of life (HRQoL) and cosmetic results in order to provide more convincing therapeutic results.

**Methods:**

Between January 2021 and June 2021, 100 cN0 patients who had a confirmed diagnosis of papillary thyroid microcarcinoma were randomized to undergo either MGTET (*n* = 50) or GTET (*n* = 50). These two groups' baseline characteristics, intraoperative and postoperative findings, were compared. The Patient and Observer Scar Assessment Scale (POSAS) was determined 6 months after surgery. Thyroid Cancer‐Specific Quality of Life Questionnaire was used to assess HRQoL at 1, 3, 6, and 12 months after surgery.

**Results:**

M‐GTET was associated with a larger number of lymph nodes dissected (*p* < 0.001), lower drainage volume (*p* < 0.001), shorter hospital stay (*p* < 0.001), and shorter axillary incision (*p* < 0.001). POSAS was more favorable in M‐GTET. HRQoL was significantly better for MGTET in terms of less problems with scar (*p* < 0.001).

**Conclusion:**

Our study suggests that MGTET provides better therapeutic, cosmetic, and HRQoL outcomes.

## 1 INTRODUCTION

In 2020, out of all cancer combined, newly diagnosed thyroid cancer accounted for 3% and 0.4% of new deaths.^1^ Thyroid cancer is the 11th most common cancer and the fifth most common cancer in females, as the incidence rate in women is three to four times higher than in men.^1^, ^2^ Papillary thyroid carcinoma is the most common form of thyroid cancer, and its primary treatment consists mainly of surgical thyroidectomies. The number of thyroid nodules detected by ultrasound examination has continuously risen during the last century. It is estimated that a thyroid nodule is present in 5% of the female population.^3^ For patients who require surgery, conventional open thyroidectomy has been the most performed surgical technique.^4^ The surgery can range from lobectomy to total thyroidectomy accompanied by central and lateral neck lymph nodes dissection. The number of thyroidectomies performed has increased due to better diagnostic techniques that detect small malignant nodules.^5^ Papillary thyroid microcarcinoma (PTMC) is called papillary thyroid carcinoma 1 cm or less, and the incidence of PTMC has dramatically increased these recent years.^6^, ^7^ Since an essential percentage of patients want a better cosmetic result, they inquire about an alternative approach other than conventional open thyroidectomies.

The first endoscopic thyroidectomy was performed in 1997 by Huscher.^8^ Over the years, many different techniques have been developed, and the most commonly used ones are the gasless trans‐axillary endoscopic thyroidectomy (GTET), bilateral axilla‐breast approach, retro‐auricular facelift approach, and trans‐oral endoscopic thyroidectomy via vestibular approach.^9^ GTET provides both a safe and reasonable cosmetic satisfaction^10^; it can only be performed for N0 patients as lymph nodes in the poststernal region cannot be dissected due to limitation in the visual field.^11^ We developed a modified approach for gasless trans‐axillary endoscopic thyroidectomy (M‐GTET).

From the patient's point of view, postoperative scar and health‐related quality of life (HRQoL) are essential. Several HRQoL scoring methods have been used to assess patients with papillary thyroid carcinoma.^12^, ^13^, ^14^ In the past few years, the cosmetic results of trans‐axillary endoscopic thyroidectomy have shown to be better than those of open thyroid surgery.^15^, ^16^, ^17^

The aim of this study is to test whether M‐GTET provides better surgical outcomes than GTET in terms of cosmetic results and HRQoL. For this purpose, we compared the postoperative findings, Patient and Observer Scar Assessment Scale (POSAS) and Thyroid‐Cancer Specific Quality of Life Questionnaires of MGTET and GTET.

## 2 PATIENTS AND METHODS

**FIGURE 1:**
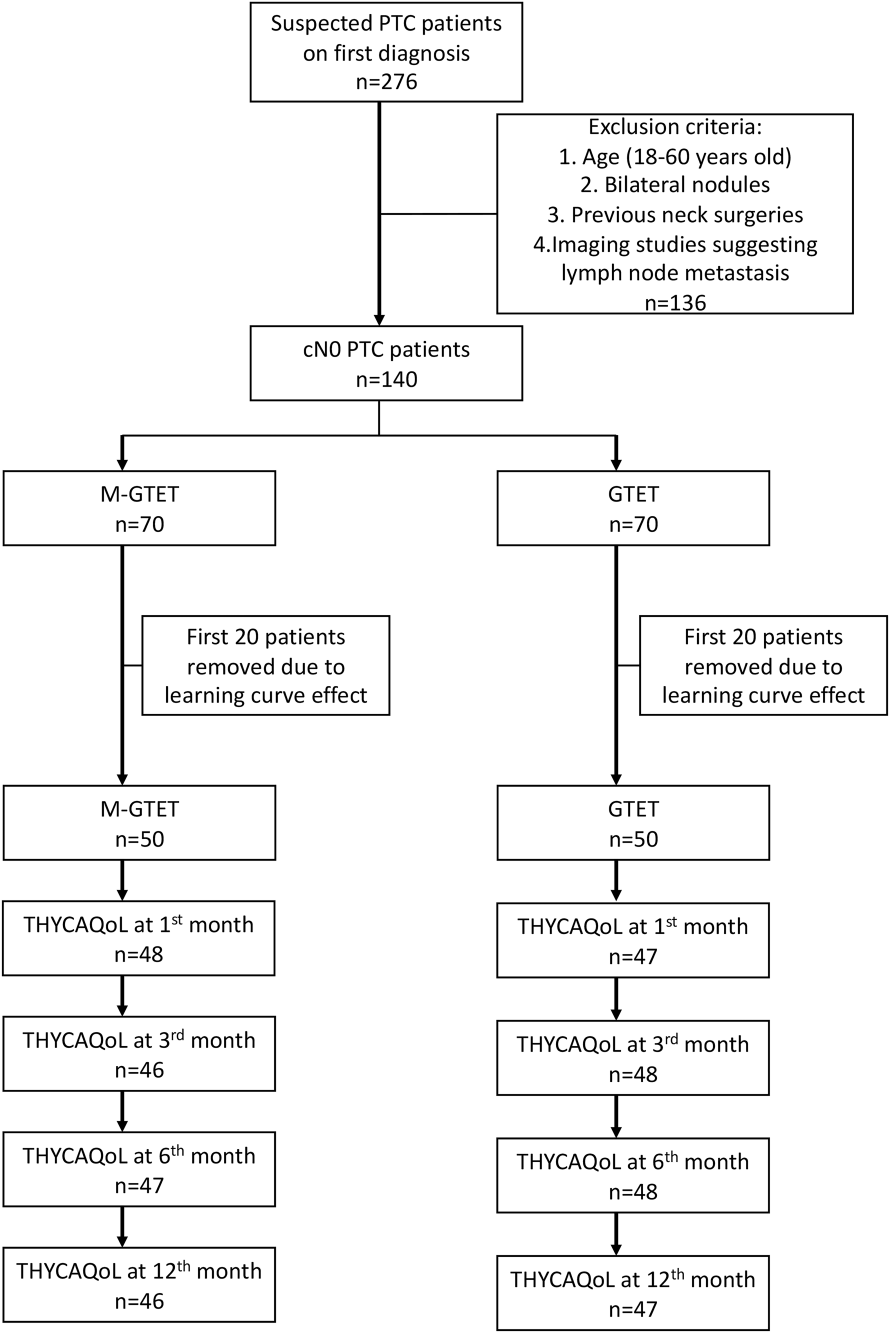
Flowchart of the study design.

This study started in January 2021, and by June 2021, 276 patients presented at the outpatient department with suspected diagnosis of PTMC. Both types of operation had the same selection criteria: no history of neck surgery, age between 18 and 60 years old, and all patients who underwent an ultrasound‐guided fine needle aspiration suspecting papillary thyroid carcinoma with unilateral malignant nodule <1 cm. The central and lateral lymph nodes were assessed by ultrasound and contrast medium computed tomography before surgery, and any patients who suspected lymph node metastasis were excluded. Patients were 1:1 randomized to the two groups. The 1:1 randomization technique was used to divide the participants into two groups: one participant was assigned to the GTET group, and the next was assigned to the MGTET group regardless of their baseline characteristics. This was done to avoid the surgeons getting more prone to perform one surgical technique than the other. The surgical morbidity of both techniques was assumed to be the same during the study. All the operations were performed by one surgeon (SWZ). 140 patients underwent GTET or M‐GTET. The first 20 cases of both GTET and MGTET were excluded from the analysis to diminish the learning curve effect, and 50 (50%) patients had GTET, and 50 (50%) patients had undergone M‐GTET. All patients had a follow‐up of at least 12 months postsurgery. Only patients with PTMC were selected for our study since 90% of patients newly diagnosed with thyroid nodules are clinically insignificant,^18^ and out of the remaining 10% of patients requiring thyroidectomies, 80%–90% are with malignant nodules of 1 cm or less.^7^ The ethics committee of Tongji Hospital approved this study, and all the patients signed informed consent forms. All the procedures involving human participants in this study were per the Declaration of Helsinki.^19^ GTET and M‐GTET were compared based on baseline characteristics, intraoperative and postoperative findings, POSAS and for HRQoL at the first, third, sixth, and 12th month after surgery. Before beginning the scar assessment, two of our team members were assigned to learn how to use the POSAS and practiced on the participants chosen to diminish the learning curve. Both evaluators were unaware of which of the participants were assigned to GTET and MGTET and did not have access to the assessment scores of the other evaluator. The average mean value of the two evaluators was used. Up to two reminders were sent to nonresponders depending on their preferred communication methods (Figure 1).

## 3 SURGICAL TECHNIQUE

### 3.1 A traditional approach

The traditional approach was performed as described in previous studies.^10^, ^20^, ^21^

### 3.2 A modified approach (Figure 2)

**FIGURE 2:**
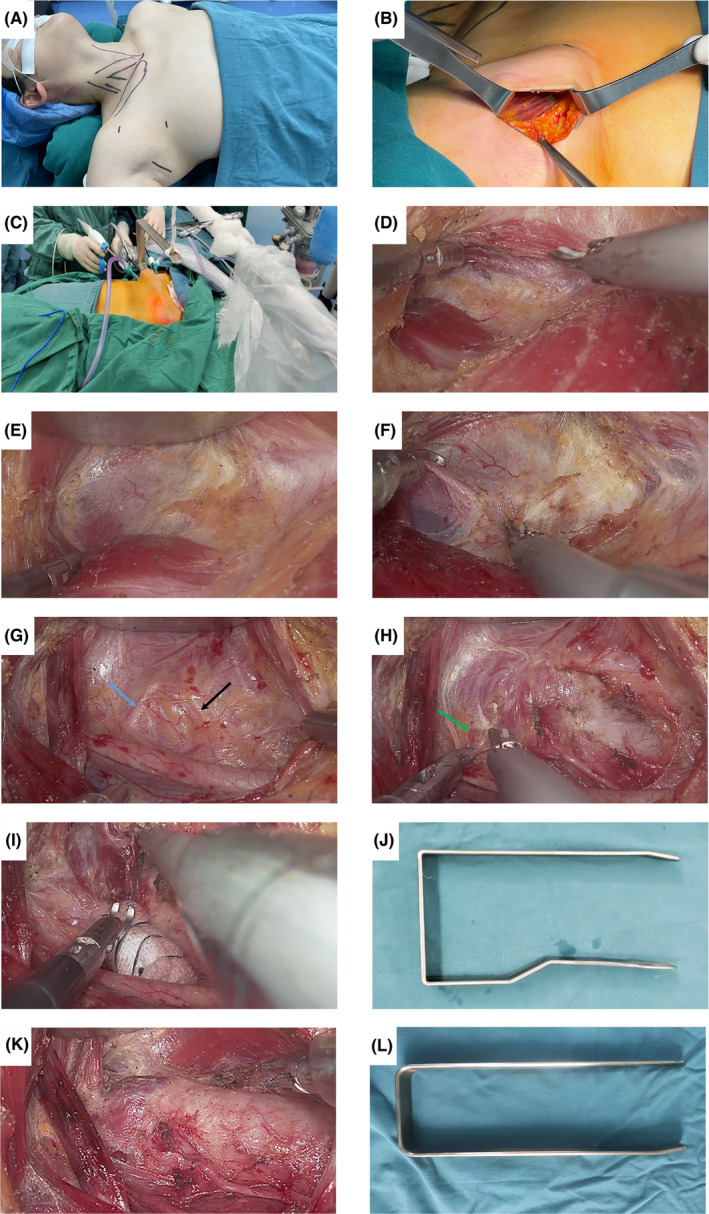
Modified gasless trans‐axillary endoscopic thyroidectomy. (A) Patient positioning and incision markings. (B) The lateral border of the pectoralis major muscle is exposed. (C) Positioning of the trocars and modified retractor during surgery. (D) Sternal and clavicular heads of the sternocleidomastoid muscle (SCM). (E) The sternal head of the SCM is lifted. (F) Exposure of the omohyoid muscle. (G) Exposure of the recurrent laryngeal nerve (black arrow) and inferior thyroid artery (blue arrow). (H) Exposure of the superior parathyroid gland (green arrow). (I) Protecting the recurrent laryngeal nerve using a wet gauze. (J) Modified version of the retractor. (K) Postresection of the thyroid gland and central neck lymph nodes. (L) Traditional version of the retractor.

The patient was placed in a supine position with one arm extended at <90° to expose the incision site at the axilla. An incision site is chosen at an appropriate natural axillary fold without exceeding the anterior axillary line. The positions of two 5 mm trocars are 2 cm exterior to the midclavicular line—one inferior to the clavicle and the second 5 cm below the first (A).

The surgical site is disinfected and draped accordingly, an incision is made, and subcutaneous fat is cut to expose the upper lateral border of the pectoralis major muscle (B). The fascia on the pectoralis major is preserved, and the surgical cavity is extended using a long head electric knife to exceed the premarked position of the two trocars by 2 cm. The modified retractor is placed in the surgical cavity from the axillary incision site, and the two trocars are inserted at the premarked positions. The video camera is inserted from the axillary incision, and one 5‐mm instrument is placed in each trocar (C). An ultrasonic scalpel is used to extend the subcutaneous cavity. The exterior jugular vein and the supraclavicular nerve should be carefully protected during this step. Injury to the supraclavicular nerve will cause numbness around the supraclavicular area. The position of the retractor is constantly adjusted to facilitate the exposure of the sternocleidomastoid muscle. The sternal and clavicular heads of the sternocleidomastoid muscle are identified, and the two bundles of tissues are separated along their natural gap (D). The retractor is adjusted to lift the sternal head of the sternocleidomastoid muscle (E). The omohyoid muscle is then exposed, followed by the internal jugular vein. The omohyoid muscle is preserved, and the strap muscle inferior to the omohyoid muscle is exposed (F). The lateral border of the strap muscle is exposed, and precautions are taken to prevent injury to the internal jugular vein and carotid artery. At this point, the thyroid gland will be visible.

The retractor is adjusted to lift the strap muscle and part of the thyroid gland, and the central neck lymph nodes are exposed (G). The recurrent laryngeal lymph node is exposed through blunt separation. The inferior thyroid artery and the inferior parathyroid glands are identified during this step. The pathway of the recurrent laryngeal nerve to its entry into the larynx is bluntly separated. The inferior thyroid artery and middle thyroid vein are coagulated at multiple points and dissected. The borders of central neck lymph nodes range from the medial edge of the carotid artery, the suprasternal fossa, the prevertebral fascia, and the midline of the trachea. The central lymph nodes are dissected along their lateral, lower, deep, and middle borders, thus performing en bloc resection. For inferior parathyroid glands that are difficult to preserve in situ, auto‐transplantation in the upper lateral edge of the pectoralis major muscle is performed and marked with nonabsorbable sutures,^22^ after proper identification of the parathyroid gland.^22^, ^23^

The isthmus is exposed by separating the strap muscle from the thyroid gland and dissected. The retractor is further adjusted to expose the superior pole to the thyroid gland. The thyroid gland's superior blood vessels are coagulated at multiple points and dissected. Care is taken to avoid injury to the superior laryngeal nerve. At this point, we can identify the superior parathyroid gland (H), and a “decapping” method of the superior pole of the thyroid gland allows careful preservation of the parathyroid glands' fascia and blood vessels.

At this point, the suspensory ligaments of the thyroid remain to be processed. A wet rolled gauze protects the recurrent laryngeal nerve (I), and the suspensory ligaments are progressively dissected. The thyroid gland, along with the central neck lymph nodes, is placed in a specimen bag and removed from the incision at the axilla to reduce the risk of thyroid and tumor implantation.^24^ A closed negative pressure drain was placed on every patient. All patients had intraoperative nerve monitoring during the surgery.^25^

The differences between the traditional and modified approaches: (1) A better‐concealed incision further away from the anterior axillary line for the modified approach. (2) A smaller incision at the axilla using a retractor we designed. (3) Two trocar incisions were made for the modified approach; on the contrary, only one trocar is placed inferior to the axillary incision for the traditional approach.

### 3.3 Outcomes measured

Pathological reports were reviewed to obtain the number of dissected and metastasized central neck lymph nodes. The total operation time was recorded from the time of incision to the closure of the incision. Skin flap preparation time was from the time of incision to just before using the video camera to pursue the surgery. The total drainage volume was obtained by readings on the drainage flask. All patients had an electro‐laryngoscope before surgery and on Day 3 postsurgery to assess the recurrent laryngeal nerve. Reduction of vocal cord movement was recorded as temporary laryngeal nerve palsy. For patients with transient laryngeal nerve palsy, the latter underwent a further electro‐laryngoscope 6 months postsurgery to assess for permanent laryngeal nerve palsy. The serum calcium levels of all participants were tested 2 days postsurgery; no participants reported hypocalcemia.

The scar of patients was assessed 6 months after surgery using the POSAS.^26^, ^27^ This scale consists of a patient part and an observer part. The patient and observer parts consist of six items and five items, respectively, scored numerically from 1 to 10, with 10 being the worst imaginable scar or sensation.

The Chinese version of the Thyroid Cancer‐Specific Quality of Life Questionnaire was used to assess for the thyroid‐specific symptoms caused by the surgery.^28^ The questionnaire consists of seven multi‐item scales (neuromuscular, voice, concentration, sympathetic, throat/mouth problems, psychological, and sensory) and six single‐item scales (problems with scar, felt chilly, tingling hand/feet, gained weight, headache, and less interest in sex). The pointing system consist of a four‐point scale ranging from 1= “not at all” to 4 = “very much,” which are linear transformed to a 0–100 scale. The higher the score, the more the complaints.

### 3.4 Statistical analysis

The software SPSS (version 24.0, IBM, Chicago, IL, USA) was used for the statistical analysis. The results were expressed as mean and standard deviation or as the number of patients where appropriate. For nonnormally distributed continuous and ordinal data, the Mann–Whitney *U* test was used. For normally distributed data, the independent samples *t*‐test was used. A two‐sided *p* < 0.05 was considered statistically significant.

## 4 RESULTS

### 4.1 Baseline characteristics

Both MGTET and GTET groups had 50 patients randomized into their respective groups. Of the 100 patients, 95/100 (95%), 94/100 (94%), 95/100 (95%) and 93/100 (93%) responded to the surgery at 1, 3, 6, and 12 months postoperatively, respectively. Figure 1 shows the flowchart of the study design. The two groups did not differ in terms of baseline characteristics. Table 1 shows the baseline characteristics of MGTET and GTET.

**TABLE 1:** Comparison of the baseline characteristics between modified gasless trans‐axillary endoscopic thyroidectomy (M‐GTET) and gasless trans‐axillary endoscopic thyroidectomy (GTET).

	M‐GTET (*n* = 50)	GTET (*n* = 50)	*p*‐value
Age, years			
Median (IQR)	31 (25–36.2)	28 (24.75–34.25)	0.317^a^
Sex (%)			
Female	82	84	0.790^b^
Male	18	16	
Ethnicity (%)			
Han	94	96	0.646^b^
Others	6	4	
BMI (kg/m^2^)			
Median (IQR)	23.7 (22.1–25.7)	23.4 (22.1–26.3)	0.855^a^
Employment status (%)			
Employed/Student	84	92	0.469^b^
Unemployed	12	6	
Retired	4	2	
Marital status (%)			
Single/Widowed/Divorced	54	56	0.841^b^
Married or in a relationship	46	44	
Smoking history (%)			
Yes	14	18	0.585^b^
No	86	82	

^a^ Derived by the Wilcoxon rank sun test.

^b^ Derived by the Pearson chi‐squared test.

### 4.2 Operative findings

Table 2 compares intra‐ and postoperative findings. The total time for MGTET and GTET was 90.7 and 97.6 min (*p* = 0.960), respectively. And there was no statistically significant difference between the operation time after skin flap preparation between MGTET and GTET (77.9 vs. 84.8, *p* = 0.930). There was a more significant number of lymph nodes dissected for the MGTET than those dissected by GTET (6.16 vs. 3.74, *p* < 0 001). However, there was no difference in the number of metastasized lymph nodes between the two groups (*p* = 0.474). No postoperative hematoma was found in both GTET and MGTET. The amount of intraoperative blood lost was statistically significantly lower for MGTET (13.8 vs. 17.2 mL, *p* < 0 001), and MGTET had a shorter postoperative hospital stay (3.38 vs. 4.24 days, *p* < 0 001). The drainage volume was statistically significantly lower in the MGTET group (89.8 vs. 104.4 mL, *p* < 0.001). No patients suffered from permanent recurrent laryngeal nerve palsy. There was no statistically significant difference in the number of patients with postoperative infections and numbness in the supraclavicular region. The length of the axillary incision was statistically significantly shorter for MGTET (32.4 vs. 46.3 mm, *p* < 0.001). The incision of M‐GTET was further away from the anterior axillary line (9.0 vs. 3.4 mm, *p* < 0.001). Since all patients had a unilateral thyroidectomy and central lymph node dissection, no patients suffered from hypoparathyroidism.

**TABLE 2:** Comparison of intraoperative and postoperative findings between modified gasless trans‐axillary endoscopic thyroidectomy (M‐GTET) and gasless trans‐axillary endoscopic thyroidectomy.

	M‐GTET (*n* = 50)	GTET (*n* = 50)	*p*‐value
Total operation time/min (SD)	90.7 (6.4)	97.6 (6.1)	0.960
Operation time after skin flap preparation/min (SD)	77.9 (5.8)	84.8 (6.0)	0.930
Blood loss/mL (IQR)	13.8 (12–16)	17.16 (16–18)	**<0.001**
Postoperative hospital stays/days (IQR)	3.38 (3–4)	4.24 (4–5)	**<0.001**
Drainage volume/mL (IQR)	89.8 (85.5–94.0)	104.4 (98.0–110.0)	**<0.001**
Number of CLN dissected (IQR)	6.16 (5–7)	3.74 (3–4)	**<0.001**
Number of CLN metastasized (IQR)	0.22 (0–0)	0.18 (0–0)	0.936
Temporary RLN palsy (%)	1 (2)	4 (8)	0.169
Permanent RLN palsy (%)	0	1 (2)	0.315
Postsurgical infection (%)	1 (2)	2 (4)	0.558
Numbness in supraclavicular region (%)	3 (6)	9 (18)	0.065
Length of axillary incision/mm (IQR)	32.4 (31–34)	46.3 (45–48)	**<0.001**
Distance of incision from anterior AL/mm (IQR)	9.0 (8–10)	3.4 (3–4)	**<0.001**

The significant values appear in bold.

Abbreviations: CLN, central neck lymph nodes; IQR, interquartile range; RLN, recurrent laryngeal nerve; SD, standard deviation.

### 4.3 Patient and observer scar assessment

Table 3 summarizes the POSAS. Out of five domains in the observer scar assessment scale, three domains had statistically significantly better scores for M‐GTET, namely vascularization, pigmentation, and thickness. Four out of six domains of the patient scar assessment scale had statistically significantly better scores for M‐GTET. The summary score of PSAS and OSAS were both more favorable for M‐GTET.

**TABLE 3:** Comparison of mean scores (95% CI) of patient and observer scar assessment scale between modified gasless trans‐axillary endoscopic thyroidectomy (M‐GTET) and gasless trans‐axillary endoscopic thyroidectomy (GTET) 6 months postsurgery.

	M‐GTET (*n* = 47)	GTET (*n* = 48)	*p*‐value^a^
OSAS summary score	19 (18.3–19.1)	21.5 (20.7–21.8)	**<0.001**
Vascularization	4 (3.6–4.1)	5 (4.6–5.1)	**<0.001**
Pigmentation	4 (3.4–3.8)	4 (4.1–4.6)	**<0.001**
Thickness	4 (3.5–3.9)	5 (4.3–4.7)	**<0.001**
Relief	4 (3.8–4.2)	4 (3.7–4.2)	0.897
Pliability	4 (3.4–3.7)	4 (3.3–4.7)	0.831
PSAS summary score	21 (20.8–21.8)	25 (24.8–25.7)	**<0.001**
Is the scar painful?	4 (3.9–4.3)	4 (3.9–4.4)	0.936
Is the scar itching?	4 (3.7–4.1)	4 (3.8–4.1)	0.517
Is the color of the scar different?	3 (3.1–3.4)	5 (4.4–4.8)	**<0.001**
Is the scar stiffer?	4 (3.4–3.8)	5 (4.5–4.8)	**<0.001**
Is the thickness of the scar different?	3 (3.0–3.4)	4 (3.6–3.9)	**<0.001**
Is the scar irregular	3 (3.1–3.4)	4 (4.0–4.4)	**<0.001**

The significant values appear in bold.

Abbreviations: OSAS, observer scar assessment score; PSAS patient scar assessment score.

^a^ Mann–Whitney *U* test.

### 4.4 Thyroid cancer‐specific quality of life

**FIGURE 3:**
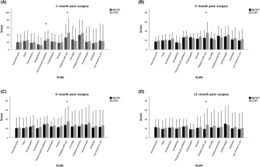
THYCA‐QoL score comparison between patients in the MGTET group and GTET group. (A) Two scales had significantly lower scores in the MGTET group 1 month postsurgery. (B–D) One scale had significantly lower score in the MGTET group at 3, 6, and 12 months postsurgery. *p < 0.05.

Table 4 and Figure 3 show the mean (standard deviation) scores of HRQoL. For the first month postsurgery, MGTET patients had significantly less throat and mouth problems (23.87 vs. 28.55, *p* = 0.026) and less problems with scar (32.30 vs. 44.94, *p* < 0.001). At 3, 6, and 12 months postsurgery, MGTET continued to register significantly lower scores for scar problems. This indicates that MGTET patients complained less about their scars.

**TABLE 4:** Comparison of health‐related quality of life in patients who underwent modified gasless trans‐axillary endoscopic thyroidectomy (M‐GTET) and gasless trans‐axillary endoscopic thyroidectomy (GTET) by using THYCA‐QoL at 1, 3, 6, 12 months postsurgery.

	First month			Third month			Sixth month			Twelfth month		
THYCA‐QoL	MGTET	GTET	*p*‐value	MGTET	GTET	*p*‐value	MGTET	GTET	*p*‐value	MGTET	GTET	*p*‐value
Neuromuscular	19.43 (10.56)	20.36 (10.51)	0.671	16.96 (9.88)	18.56 (10.44)	0.464	20.60 (11.15)	22.00 (10.76)	0.508	22.72 (8.17)	21.52 (8.66)	0.498
Voice	22.64 (12.81)	24.74 (13.08)	0.418	19.48 (11.41)	21.15 (11.31)	0.542	20.45 (10.70)	22.57 (10.82)	0.339	18.67 (9.28)	21.22 (9.29)	0.127
Concentration	18.00 (12.11)	19.43 (11.80)	0.538	19.85 (12.11)	23.48 (12.06)	0.143	23.64 (9.80)	26.53 (10.85)	0.168	21.83 (10.60)	22.33 (9.62)	0.700
Sympathetic	13.79 (11.35)	11.32 (10.15)	0.295	15.26 (14.10)	14.13 (12.83)	0.697	20.40 (9.55)	22.91 (9.71)	0.165	19.41 (9.71)	22.30 (8.98)	0.106
Throat/mouth problems	23.87 (11.77)	28.55 (10.92)	**0.026** *	26.07 (13.39)	27.98 (11.76)	0.425	28.55 (7.49)	30.66 (7.92)	0.172	23.91 (7.80)	25.83 (6.65)	0.232
Psychological	21.51 (10.72)	20.87 (9.66)	0.747	26.54 (7.89)	24.54 (9.88)	0.260	23.98 (5.69)	25.68 (8.14)	0.280	18.02 (6.16)	18.41 (5.78)	0.730
Sensory	17.60 (10.27)	19.60 (11.04)	0.390	23.09 (11.52)	27.48 (11.86)	0.110	22.55 (9.64)	25.40 (9.25)	0.135	20.93 (11.46)	22.76 (11.38)	0.494
Problems with scar	32.30 (17.53)	44.94 (19.97)	**<0.001** *	24.74 (15.85)	35.87 (22.89)	**0.013** *	27.38 (17.30)	35.11 (16.00)	**0.033** *	18.65 (16.54)	27.98 (16.99)	**0.011** *
Felt chilly	26.68 (17.73)	23.17 (16.74)	0.229	24.24 (13.45)	21.98 (12.29)	0.382	23.87 (16.43)	27.38 (15.87)	0.323	22.24 (15.64)	25.83 (16.92)	0.280
Tingling hand and feet	39.32 (20.22)	44.23 (17.24)	0.197	29.41 (19.96)	26.54 (19.20)	0.471	24.57 (16.09)	28.79 (16.31)	0.204	21.52 (15.89)	22.96 (16.86)	0.598
Gained weight	20.36 (18.91)	22.47 (19.60)	0.607	27.98 (19.64)	31.57 (19.52)	0.429	28.09 (18.18)	32.30 (18.83)	0.243	30.13 (18.18)	27.26 (21.22)	0.471
Headache	16.85 (19.31)	18.96 (17.87)	0.583	21.52 (18.69)	24.39 (22.47)	0.511	21.77 (17.24)	25.28 (17.15)	0.359	20.09 (16.28)	22.96 (15.35)	0.439
Less interest in sex	23.87 (22.51)	25.28 (20.89)	0.761	19.37 (19.15)	22.24 (20.93)	0.499	23.17 (18.09)	26.68 (16.34)	0.341	18.72 (16.47)	20.80 (16.10)	0.530

The significant values appear in bold.

* *p* < 0.05.

## 5 DISCUSSION

The advantages of M‐GTET over GTET include: (1) Less collision between instruments due to the two entry points of the trocars being further apart than in GTET. The clash of instruments in endoscopic thyroidectomies is due to the small cavity of the workspace and trocars being too close to each other, limiting the surgeon's movement.^29^ (2) The modified retractor reduces the tension of the skin at the axillary incision. Since the 30° endoscope used for surgeries is always positioned at the lower border of the axillary incision, the middle and upper boundary of the incision is left as “dead space.” The modified version of the retractor helps reduce the amount of “dead space.” (3) Better identification and protection of the parathyroid glands. The placement of the two trocars helps to provide a better view of the post sternal lymph nodes and thymus gland. Since about 38% of lower parathyroid glands are located in the thymus gland,^30^, ^31^ better protection of the thymus gland pertains to better protection of the inferior parathyroid glands. “Decapping” of the upper thyroid pole helps protect the superior parathyroid gland by preserving its surrounding fascia and blood vessels. (4) Less friction between the instruments and pectoralis major muscle. In GTET, friction between the instruments and the pectoralis major muscle causes unnecessary wear and tear, which can cause excessive damage to the muscle tissue and bleeding, showed by less bleeding in MGTET. Placing two trocars on both sides of the retractor could overpass the obstruction caused by the cervical bone for central lymph node dissection. The two trocars in the modified approach can be considered pivot points for the instruments. Thus, the pivot point for the traditional approach is almost horizontal with the clavicle. Performing central lymph node dissection with the traditional approach proved more time‐consuming and challenging as the clavicle is an obstacle. The clavicle can obstruct the surgeon from grabbing and dissection level VIA and VIB central neck lymph nodes. On the other hand, the pivot points for the modified approach were at a higher position to the clavicle. Our surgeon, SWZ, found it was more convenient to perform central lymph node dissection using the modified approach. Furthermore, our surgeon found it was more convenient to manipulate the instruments when placed in trocars as in the modified approach, as the latter serves as a leverage point; manipulating and controlling the instrument placed together with the camera at the axillary incision in the traditional approach can be difficult and tiring at times as there is no leverage point. MGTET was proved to provide a thorough dissection of the lymph nodes, although there was no difference between the number of metastasized lymph nodes, and this is because, before surgery, all patients had central lymph nodes assessed by ultrasound and CT of the neck.^32^, ^33^ The drainage volume for MGTET was significantly lower, which may be due to less frictional damage to the pectoralis major muscle, thus pertaining to shorter mean hospital stay that were recorded for MGTET. We firmly believe that M‐GTET provides a better poststernal dissection of central lymph nodes, thus providing better therapeutic care. Moreover, the modified version of the retractor reduces the unnecessary tension at the site of the incision, thus decreasing the “dead space.” Lower drainage volume and shorter hospital stays are also advantages of M‐GTET. At the time of writing, all patients have been followed up on a regular basis, and no signs of relapse were detected in both GTET and MGTET.

The modified version of the retractor enabled to have a shorter incision at the axilla and at the same time being further from the anterior axillary line for the MGTET group. This helps to provide better concealment of the incision. Moreover, the modified retractor helps to provide less overextension of the skin at the axilla during the surgery, without affecting the field of view. Due to less over‐extension of the incision, and although placing two 5 mm trocars, the POSAS had better scores for MGTET.

Treating the disease and at the same time a patient's overall well‐being is also very important. Several studies have mentioned that scarring can affect the QoL of patients.^34^ The importance of QoL of patients was also mentioned in the recent American Thyroid Association Guidelines as the choice of surgery should take into consideration QoL's long‐term effect.^3^ Scarring problems can affect the daily life of patients ranging from difficult choice of clothes, loss of confidence, and sense of being secluded.^35^ Thus, MGTET is a better option for PTMC patients who have esthetic needs.

## 6 CONCLUSIONS AND LIMITATIONS

Our current study demonstrates that for patients eligible for unilateral endoscopic thyroidectomies, MGTET is the better option in terms of better therapeutic outcomes, better POSAS scores, and less complaints about scars. Our study was a single‐centered experience and single‐surgeon comparison between M‐GTET and GTET. Our study is not without limitations: Multicenter and multisurgeon studies should be carried out to validate our study further. Furthermore, comparison between MGTET and open thyroidectomies and other endoscopic techniques is needed to obtain a more in‐depth validity of our modified approach.

## AUTHOR CONTRIBUTIONS

**Deenraj Kush Dhoomun:** Conceptualization (lead); data curation (lead); investigation (lead). **HuiLan Cai:** Conceptualization (supporting). **Ning Li:** Methodology (supporting). **YanHuan Qiu:** Investigation (supporting). **XingRui Li:** Supervision (equal); validation (equal). **XiaoPeng Hu:** Supervision (equal); writing – original draft (equal). **WenZhuang Shen:** Conceptualization (lead); methodology (lead); supervision (equal).

## CONFLICT OF INTEREST STATEMENT

The authors declare that there is no conflict of interest.

## ETHICS STATEMENT

Ethics approval for this study was obtained by the ethics committee of Tongji Hospital (TJ‐C20180110), and all the patients involved signed informed consent forms. All the procedures involving human participants in this study were per the Declaration of Helsinki.

Signed patient approval was obtained to produce photographs.

## Data Availability

All data are available upon request to the corresponding author.
